# The scope and strength of sex-specific selection in genome evolution

**DOI:** 10.1111/jeb.12201

**Published:** 2013-07-13

**Authors:** A E Wright, J E Mank

**Affiliations:** * Department of Zoology, University of Oxford, Edward Grey InstituteOxford, UK; † Department of Genetics, Evolution and Environment, University College LondonLondon, UK

**Keywords:** sex chromosomes, sex-biased gene expression, sex-specific selection, sexual dimorphism

## Abstract

Males and females share the vast majority of their genomes and yet are often subject to different, even conflicting, selection. Genomic and transcriptomic developments have made it possible to assess sex-specific selection at the molecular level, and it is clear that sex-specific selection shapes the evolutionary properties of several genomic characteristics, including transcription, post-transcriptional regulation, imprinting, genome structure and gene sequence. Sex-specific selection is strongly influenced by mating system, which also causes neutral evolutionary changes that affect different regions of the genome in different ways. Here, we synthesize theoretical and molecular work in order to provide a cohesive view of the role of sex-specific selection and mating system in genome evolution. We also highlight the need for a combined approach, incorporating both genomic data and experimental phenotypic studies, in order to understand precisely how sex-specific selection drives evolutionary change across the genome.

The ability to attract mates and reproduce is a central component of Darwinian fitness. As well as primary sexual dimorphisms directly involved in reproduction, including numerous gametic proteins key to syngamy, there are many secondary sexual traits involved in mate acquisition (Andersson, 1994; Swanson & Vacquier, 2002; Nadeau *et al*., 2007). Selection related to sex can be a powerful force given that it is a crucial component in Darwinian fitness, and it can act in opposite directions for males and females due to their distinct reproductive roles and biology (Arnqvist & Rowe, 2005). Aside from sex-limited regions, such as Y or W chromosomes, contradictory female- and male-specific selection regimes act on a genome that is shared between the sexes.

Sex-specific selection is strongly influenced by mating system (Fig.1), which also affects neutral diversity and evolution. At one end of the spectrum, the potential for conflict between female- and male-specific selection is lowest in monogamous systems. In these species, sex-specific selection often acts primarily on reproductive biology, resulting in very few pronounced secondary sexual dimorphisms (Helfenstein *et al*., 2004). Promiscuous systems show much more potential for sexual conflict (Lifjeld *et al*., 1993; Lindenfors *et al*., 2002; Arnqvist & Rowe, 2005). Polyandrous and polygynous species are often characterized by large differences between male and female parental effort and other aspects of life histories. This creates ample scope for sex-specific selection (Fig.1) (Kokko *et al*., 2012). Mating system also influences neutral diversity and rates of evolution. Large differences in the variance in reproductive success between males and females increase the rate of genetic drift, the strength of which varies across the genome depending on the degree and direction of sexual asymmetry in inheritance (Vicoso & Charlesworth, 2009; Mank *et al*., 2010c). Contrasting evolutionary rates and diversity among these regions, most often between the sex chromosomes and the autosomes, can be used to infer the power of neutral evolutionary forces at work in the genome, as well as estimate mating system (Corl & Ellegren, 2012).

**Figure 1:**
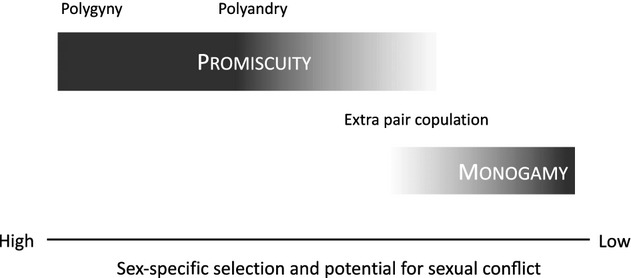
Mating systems and sex-specific selection. Sex-specific selection is strongly influenced by mating system. The potential for conflict between female- and male-specific selection is lowest in monogamous systems. In mating systems with large differences in reproductive potential, the divergence in male and female fitness optima is greater, as is the probability that sex-specific selection forces are contradictory. As a result, the potential for sexual conflict is predicted to increase with the magnitude of difference in mating success. Additionally, polyandrous and polygynous species are often characterized by large differences between male and female parental effort and other aspects of life histories, which creates ample scope for sex-specific selection. Thus, sex-specific selection and sexual conflict may play a more significant role in polygnous and polyandrous mating systems than monogamous systems.

Conflicting sex-specific selection has the potential to create a significant evolutionary burden on a population (Foerster *et al*., 2007; Morrow *et al*., 2008; Connallon *et al*., 2010), and resolving this conflict, when it is possible at all, both relieves this burden and allows the sexes to reach separate fitness optima (Chapman *et al*., 2003). Phenotypic-level studies have revealed a significant level of conflict in many organisms (Chippindale *et al*., 2001; Arnqvist & Rowe, 2002; Magurran & Seghers, 1994, among many others), affecting many different phenotypes.

Recent genomic and transcriptomic studies have made it possible to take studies to the molecular level, and assess the magnitude, locus, and resolution of conflicting sex-specific selection (Innocenti & Morrow, 2010; Moghadam *et al*., 2012). These studies indicate that sex-specific evolutionary forces, both adaptive and neutral, affect a large proportion of the genome (Connallon *et al*., 2010) and play a significant role in determining rates of both adaptive and neutral change for gene sequence, gene expression and genome structure. This is reinforced by the disproportionately large effect that sexually antagonistic loci are predicted to have on genetic variation for fitness (Arnqvist, 2011; Long *et al*., 2012). Additionally, it is clear from molecular studies that there are different routes to resolving conflict between female- and male- specific selection (Connallon, 2007; Haig, 2009; Gallach & Betran, 2011).

Molecular approaches offer the promise of identifying sexually antagonistic genes and alleles, as well as creating an understanding of how discrete male and female phenotypes are encoded. However, there are a number of important assumptions made when genomic data is used in isolation to infer the nature of sexual conflict and sex-specific selection. One key assumption is that, for the phenotypic traits in question, conflict between female- and male- specific selection has been resolved through the evolution of sex-specific gene regulation or some other mechanism to break down intersexual correlation. Incorporating experimental studies in addition to genomic approaches can shed light on this fundamental assumption and provide detailed information we lack on the fitness consequences of many genomic mechanisms thought to resolve sexual conflict (Tregenza *et al*., 2006).

Despite the potential of combining phenotypic and molecular data, aside from a few exceptions (Innocenti & Morrow, 2010; Moghadam *et al*., 2012), these methods have proceeded largely independent of each other. Our purpose here is to synthesize recent molecular genomic advances in order to create a cohesive picture of the importance of sex-specific selection in shaping the evolution of various genomic properties. Ultimately, our goal is to understand how sex-specific selection and mating system affects genome evolution through adaptive and neutral processes, and reinforce the need for a combined approach, incorporating both experimental phenotypic and molecular studies, to create a cohesive understanding of sex-specific selection, its fitness consequences, genomic targets and mechanisms of resolution.

## Sex-specific selection and adaptive change

Most molecular genetic analysis of conflicting sex-specific selection focuses on intralocus conflict (conflict where an allele at a given loci is beneficial to one sex but detrimental to the other), where opposite female- and male-selection acts on the same locus. Intralocus conflict can be resolved by a number of genetic mechanisms, the most studied of which is sex-biased expression (Connallon & Knowles, 2005; Gallach & Betran, 2011), which represents the breakdown in intersexual correlation in gene regulation. Additionally, duplication of genes with sex-specific functions (Gallach *et al*., 2010) may also be important in resolving conflict. In either case, sex-biased or sex-specific gene expression is used as a signature of at least partially resolved conflict, as the breakdown of correlation between male and female transcription allows for sex-specific fitness optima (Connallon & Knowles, 2005; Mank, 2009). In contrast, it is more difficult to detect interlocus conflict (conflict between alleles at different loci, where if one allele is beneficial to males and detrimental to females, the other allele displays a reverse fitness effect) based on molecular data alone, as there is no expected resolution through the breakdown in intersexual correlation. However, imprinting and parent-of-origin expression may indicate sites linked in interlocus conflict (Haig, 2009; Gregg *et al*., 2010a,b), as well as allelic imbalance in expression, although with caveats. The genetic mechanisms by which sexual conflict can be resolved are discussed in further detail below.

### Types of genes influenced by sex-specific selection

Initial studies examining the influence of sex-specific selection at the genetic level focused on known reproductive genes (Swanson & Vacquier, 2002), which evolve rapidly across a diverse range of taxa. Many of the genetic changes have been shown to be adaptive (Swanson *et al*., 2001), with one of the best examples perhaps being the evolution of male accessory gland proteins (Acps) related to sperm competition in *Drosophila*. These proteins are present in seminal fluid, and act to increase male mating success by promoting ovulation, reducing female receptivity to remating and promoting sperm storage (Wolfner, 2002). Acp loci in *Drosophila* are 50% more divergent than nonreproductive proteins, with many exhibiting rapid turnover between species and high rates of functional change indicative of positive selection (Swanson *et al*., 2001; Begun & Lindfors, 2005). This is a broad pattern, as similar signatures of adaptive change have been found in a wider group of reproduction-related genes, including seminal fluid proteins, in *Drosophila* (Haerty *et al*., 2007), primates (Clark & Swanson, 2005) and rodents (Turner *et al*., 2008), as well as gamete recognition proteins in marine invertebrates (Metz & Palumbi, 1996; Palumbi, 1999). The rapid evolutionary rates of male reproduction-related genes suggest post-copulatory selection is important in driving adaptive divergence. Indeed, sperm competitive ability in *Drosophila* has been directly linked to polymorphism in certain male reproductive genes (Fiumera *et al*., 2005). Presumably, increasing competition between males for mating increases the intensity of sperm competition, thereby resulting in higher rates of evolution for male reproduction-related genes (Walters & Harrison, 2011). Reproductive character displacement may also contribute to the rapid divergence of male reproductive genes (Matute, 2010) as well as frequency-dependent selection (Clark *et al*., 1999).

More recently, female reproductive proteins in *Drosophila* have been shown to undergo similarly high rates of functional change (Swanson *et al*., 2004; Panhuis & Swanson, 2006), possibly due to the conflict between the sexes over polyspermy. Sperm competition generates selection on males to increase the speed of fertilization. Increased sperm fertilization rate can result in a cost to females through the elevated possibility of multiple sperm penetrating the ovum, which generally results in lethality. The major cost to females of egg inviability generates female-specific selection to slow down fertilization. ZP3, a protein found within the egg coat, is responsible for binding to sperm and thus facilitating fertilization, and there is evidence in mammals of positive selection within the gene region responsible for sperm binding (Swanson *et al*., 2001). Similar results have been shown in birds (Calkins *et al*., 2007; Berlin *et al*., 2008); however, there is some debate as to whether selection against polyspermy is responsible for driving this adaptive change, as birds may be more tolerant of polyspermy than other animals (Wishart, 1987; Birkhead & Fletcher, 1998; Tarin & Caro, 2000; Stepinska & Bakst, 2007). Instead, cryptic female choice for specific male sperm type may be responsible for the high rates of functional change seen at sperm binding regions of some egg coat proteins (Calkins *et al*., 2007; Berlin *et al*., 2008).

Male and female fitness is not solely reliant on reproductive proteins, and secondary sexual characters can play an important role in mate acquisition. As expected, there is evidence of adaptive change as a result of sex-specific selection acting on these characters. Male plumage colour in galliforms is highly diverse and shown to be involved in female mate choice and between male-to-male competition. The MC1R locus, which contributes to plumage pigmentation, has been shown to undergo high rates of functional change (Nadeau *et al*., 2007). Additionally, this rate correlates with the degree of sexual dichromatism exhibited across galliform species, demonstrating that the MC1R locus is a target for sex-specific selection acting on plumage coloration. However, many somatic dimorphisms are complex aggregates of many loci, and this complexity requires fundamentally different approaches, explained below.

### Sex-biased expression

More complex sexual phenotypes composed of dozens to hundreds of loci can be examined at the genomic scale with transcriptome data. The majority of polygenic sexual dimorphisms result from differences in gene expression between males and females, and this sex-biased expression is the product of the breakdown in intersexual correlation in expression, and therefore represent loci where intralocus conflict has been at least partially resolved (Connallon & Knowles, 2005). Additionally, rates of evolution of sex-biased genes, measured in aggregate, may offer insight into the relative strength of male- vs. female-specific selection.

In adults, the differences between males and females in expression are prevalent in the transcriptomes of many animals, including *Drosophila* (Jin *et al*., 2001; Ranz *et al*., 2003), mouse (Yang *et al*., 2006), *Anopheles* mosquitoes (Marinotti *et al*., 2006), birds (Mank *et al*., 2008a; Naurin *et al*., 2011), *C. elegans* (Cutter & Ward, 2005) and ants (Ometto *et al*., 2010). Some of the pattern of sex-bias is condition-dependent (Wyman *et al*., 2010), consistent with predictions about some types of sexually selected traits. Additionally, many of the differences in gene expression between the sexes are thought to arise from androgen- or oestrogen-mediated regulation (Zauner *et al*., 2003), and changes in regulation have produced large variation in the proportion of the transcriptome showing sex-bias among species (Zhang *et al*., 2007), as well as variation in sex-bias among populations within species (Muller *et al*., 2011; Moghadam *et al*., 2012).

Characterizing the degree of sex-biased gene expression at the tissue level can provide insight into the phenotypic traits subject to the greatest degree of sex-specific selection. As expected from studies on reproductive proteins, sex-biased expression is most evident in gonad transcriptomes (Parisi *et al*., 2004; Rinn *et al*., 2004; Mank *et al*., 2008a). However, sex-biased gene expression is observed across a large majority of somatic tissues in many animals (Yang *et al*., 2006; Mank *et al*., 2008a), particularly in the liver. Evidence from mammals and birds suggests that the degree of sex-biased expression is lowest in the brain (Yang *et al*., 2006; Mank *et al*., 2008a; Reinius *et al*., 2008). However, these studies tend to examine the brain as a whole and thus, a more detailed examination of specific areas of the brain may reveal a more obvious pattern of sex-bias (Naurin *et al*., 2011).

It is possible to estimate the relative strength of sex-specific selection at the molecular level by estimating rates of divergence for sex-biased genes. Numerous studies have found evidence of accelerated rates of evolution, particularly due to positive selection, in adult male-biased genes across a range of species (Good & Nachman, 2005; Khaitovich *et al*., 2005; Ellegren & Parsch, 2013). In addition to divergence in coding sequence, expression divergence is more pronounced for male-biased genes in *Drosophila* than female-biased genes (Meiklejohn *et al*., 2003; Llopart, 2012). However, there are exceptions to the higher rates of evolution seen in male-biased compared to female-biased genes. Some studies have found either no difference in divergence patterns between the sexes (Metta *et al*., 2006), potentially linked to a reduction in sexual selection, or the reverse pattern (Mank *et al*., 2010a). The latter study highlights the shifting nature of sex-specific selection throughout development, as female-biased genes were found to have higher rates of adaptive change when measured in the gonad during embryonic development compared to rates in adults.

Genetic architecture, the number and type of loci underlying a given trait, plays an important role in determining the genetic and phenotypic outcome of sex-specific selection and evolution of sex-biased expression. The involvement of a single gene in the development of multiple traits, pleiotropy, determines the extent to which intralocus sexual conflict can be resolved and thus, the ability of sex-specific selection to facilitate evolutionary change (Harano *et al*., 2010). Less pleiotropic genes, measured as a function of tissue specificity, exhibit sex-biased gene expression and faster rates of evolutionary change compared to pleiotropic genes (Mank *et al*., 2008b; Meisel, 2011). This may suggest that pleiotropy hinders the breakdown in intersexual correlation in expression, and therefore the capacity of the genome to respond to sex-specific selection, reinforcing the widely acknowledged role of pleiotropy as an evolutionary constraint (Fisher, 1930; Orr, 2000; Snell-Rood *et al*., 2010). Thus, the effect of selection on gene expression will not be the same between genes under different architectural constraints. Interestingly, female-biased genes display greater pleiotropic effects than male-biased genes, potentially contributing to the different rates of evolution between the two classes of genes (Assis *et al*., 2012).

Surveys of species and population variation in expression, such as those cited above, provide a long-term evolutionary view of sex-specific selection and sexual conflict. Using sex-bias expression data to infer the targets and strength of sex-specific selection relies on a number of assumptions. First, the relationship assumes that sex-biased genes encode sex-specific phenotypes and often have sex-specific fitness effects. It is difficult to test this assumption with species and population transcriptome data alone, as these data cannot directly connect large aggregates of genes with differential expression to concrete phenotypes, although there is some empirical support (Connallon & Clark, 2011a). Second, the relationship assumes that sexual conflict can be at least partially resolved via the breakdown of intersexual expression correlation; therefore, pleiotropic constraints may mask loci that are subject to sexual conflict.

Studies that combine molecular and phenotypic approaches are just now appearing, to date only two have been published to our knowledge (Innocenti & Morrow, 2010; Moghadam *et al*., 2012) and these have the added power of being able to measure sex-specific fitness effects and therefore estimate sex-specific phenotypic optima. In one case the predicted relationship between sex-biased expression and sex-specific fitness was not evident, possibly due to insufficient variation in sex-biased regulatory variation within study populations. If this is a general trend, it may suggest that short-term sex-specific regulatory changes are rare (Innocenti & Morrow, 2010). However, the other study was somewhat contradictory, and showed that changes in sex-specific selection over short evolutionary histories cause substantial changes in sex-biased expression, and that this has sex-specific fitness consequences (Moghadam *et al*., 2012). Further experimental evolution studies are planned or in progress and will no doubt provide exciting developments to this debate.

### Sex-limited expression and imprinting

The negative fitness consequences of sexual conflict (Morrow *et al*., 2008) can be avoided if antagonistic genes are sex-limited, with expression completely restricted to either males or females. Sex-limited expression, where a gene is expressed in only one sex, is somewhat different from sex-biased expression, where a gene is expressed in both females and males, but at different levels. True sex-limited expression is relatively rare for genes not linked to the sex-limited Y or W chromosomes. This may be because selection against expression in one sex decreases as expression level wanes for most loci, creating a saturation point at which sex-specific selection is not strong enough to further reduce expression. However, the point at which sex-biased expression becomes functionally sex-limited is somewhat arguable, as very low levels of expression in one sex are likely to have little phenotypic effect. This may suggest that most very sex-biased genes are functionally sex-limited.

Sex-limited genes avoid fitness penalties in the sex lacking expression (Rice, 1984). Genomic imprinting is one potential mechanism to achieve sex-limited expression without the need to breakdown intersexual regulatory mechanisms. For an imprinted allele, expression depends upon the parent of origin, and studies have suggested that imprinting can both resolve intralocus conflict and exacerbate interlocus conflict (Day & Bonduriansky, 2004; Swaney *et al*., 2007; Van Cleve & Feldman, 2007; Hager *et al*., 2008; Haig, 2009).

The resolution of intralocus conflict by the evolution of imprinting has been modelled under a wide range of selection and dominance parameters (Day & Bonduriansky, 2004; Van Cleve & Feldman, 2007; Patten & Haig, 2008). Negative mother–son fitness correlations can result in selection for invading modifier loci that silence sexually antagonistic maternally inherited alleles in males. In support of these predictions, sex-specific differences in imprinting effects of loci contributing to body size have been shown in mice (Hager *et al*., 2008).

For the majority of imprinted loci however, phenotypic effect is poorly understood, and it is possible that modelling sexual antagonism at only one locus is unrealistic. Instead, sexual conflict between interacting genes may drive the evolution of imprinting. The insulin-like growth factor 2 (Igf2) and insulin-like growth factor 2 receptor (Igf2r) are imprinted in many mammals and heavily involved in the regulation of growth (Day & Bonduriansky, 2004). This imprinting pattern of Igf2 and Igf2r is thought to be the product of conflict over maternal and paternal reproductive interests as they play out through the offspring. The expression of Igf2 is paternally inherited and associated with increased growth rates, and a mutation in this gene causes the poor growth associated with Silver–Russell syndrome (Obermann *et al*., 2004). The paternal allele of Igf2 works in concert with Igf2r, which is maternally imprinted and is responsible for degrading Igf2 (Wilkins & Haig, 2003) and limiting nutrient extraction from the mother. In addition to Igf2 and Igf2r, interlocus conflict has been implicated in the evolution of imprinting for other developmental traits such as suckling intensity and age of sexual maturation (Haig, 2009) and presumably as the phenotype of more imprinted genes is examined, the exact role of interlocus conflict will become apparent.

Recent genome-wide assessments of genomic imprinting based on parent-of-origin expression imbalanced in the offspring (Gregg *et al*., 2010a,b) indicated that a large proportion of the genome exhibits some form of imprinting, and that partial imprinting may be common for many genes, or even parts of genes. However, for many loci, the phenotypic and evolutionary effects of imprinting are unclear, and there are recent concerns regarding the accurate identification of imprinted loci (DeVeale *et al*., 2012). Further work characterizing the fitness consequences of imprinting and ascertaining whether they exhibit a sexually antagonistic pattern is key for cementing the relationship between imprinting and sexual conflict.

Despite the controversy of genome-wide evidence of imprinting, evidence from the studies referenced above suggests that imprinted loci are beacons of sexual conflict, and careful examination of numbers and rates of evolution of imprinted genes will provide insight into the strength of sexual conflict arising from sex-specific selection. However, as both inter- and intralocus sexual conflict increase, the number of imprinted loci is predicted to also increase, and further work on a larger number of sexually antagonistic imprinted loci over a range of mating systems can be used to explore this. Additionally, there is very little information about how parent-of-origin imprinting varies within and among populations, and how this regulatory mechanism responds to sex-specific selection in an experimental evolution framework.

### Post-transcriptional regulation

In addition to transcriptional differences between the sexes, post-transcriptional mechanisms also differ. Alternative splicing is a gene regulatory mechanism that produces multiple distinct transcripts from one locus, thereby increasing transcriptomic complexity without unduly adding to genome size. Sex-specific splicing is a key component of sex-specific phenotypes, such as sex determination in *Drosophila* (Telonis-Scott *et al*., 2009), and therefore, may be a general mechanism to resolve sexual conflict over gene function. Comprehensive attempts have recently been made to quantify the exact extent of sex-specific splicing throughout the genome and have shown significant conservation of sex-specific splice variants (Blekhman *et al*., 2009; Telonis-Scott *et al*., 2009; Prince *et al*., 2010).

It is easy to imagine the potential for sex-specific splicing, both in response to sex-specific selection and a route to resolve sexual conflict. Studies of alternative splicing evolution have suggested that splice variants are adaptive, and that they allow for increasing phenotypic complexity without the need to increase genome size (Barbosa-Morais *et al*., 2012; Merkin *et al*., 2012). However, aside from the role of sex-specific splicing in sex determination in insects, little is known at this point about the exact role of alternate splicing in sexually dimorphic phenotypes, or how splice variants evolve in response to sex-specific selection.

In addition to alternative splicing, sex-specific post-transcriptional regulation can be achieved via small noncoding mRNAs. Small noncoding mRNAs are thought to play an important role in development (Stefani & Slack, 2008) via both transcriptional and post-transcriptional regulation (Engels & Hutvagner, 2006). In a recent study of ten candidate small noncoding mRNAs in two *Drosophila* species, three were found to exhibit a highly male-biased expression pattern (Jiang *et al*., 2011). The extent of sex-bias was large, with one candidate expressed 40 times more in males than females. It has yet to be verified how these differences translate to the level of the phenotype, but a role in sex-specific RNA regulation seems possible. Further work on this area is needed to explore how these small noncoding RNAs regulate gene networks and phenotypic change, before inferences about the strength of sex-specific selection can be made.

### Gene duplication

In some cases, sexual conflict at a specific locus can be resolved via gene duplication. The duplication of a sexually antagonistic locus generates paralogs, which provide the potential for sex-specific neo- or subfunctionalization. Sex-specific functionalization can act on both paralogs to theoretically generate male and female beneficial duplicates, which would eventually exhibit sex-biased or sex-limited expression (Connallon & Clark, 2011b; Gallach & Betran, 2011). Alternatively, one paralog may maintain its original function, particularly when it is pleiotropic, and the resolution of sexual conflict can be achieved through sex-biased expression of the other copy.

Evidence indicates that selection to resolve sexual conflict acts to retain duplicates within the genome, and the larger the degree of conflict, the stronger the selection to resolve this conflict via the retention of a resolving duplicate. Nuclear-encoded mitochondrial genes are thought to be under sexually antagonistic selection for the rate of energy production. High rates increase the fitness of sperm but also result in a higher mutation rate, which is disadvantageous for female reproductive tissues. It has been shown among relocated duplicate genes of this class, that a large number exhibit testis-specific expression. This duplication and subsequent sex-limited expression decouples the sexes divergent fitness optima and allows resolution of the conflict over energy production (Gallach *et al*., 2010; Gallach & Betran, 2011). Recent evidence suggests that duplications are associated primarily with the evolution of male-biased not female-biased expression (Wyman *et al*., 2012); however, further experimental studies across organisms with different mating systems are necessary to determine whether this pattern is due to variation in the strength of sex-specific selection, or simply a function of the higher rate of male meiosis, and therefore origin of gene duplicates, associated with continuous sperm production.

### Beyond the autosomes

**Figure 2:**
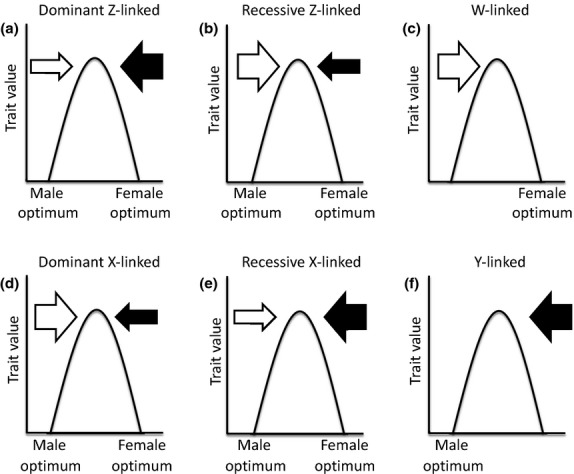
Relative strength of sex-specific selection acting on different sex chromosomes. Relative sex-specific selection is shown by arrow size, white arrows represent female-specific selection, black arrows represent selection towards male-specific optima. For dominant Z-linked alleles (panel a), male-specific selection is relatively stronger due to the fact that the Z is present more often in males than females. Recessive Z-linked alleles (panel b) are more often exposed in females to selection due to female hemizygosity; therefore, female-specific selection is relatively stronger. W-linked genes (panel c) are only selected for female-specific effects. Dominant X-linked alleles (panel d) are more often selected for female-specific effect because the X is more often present in females, whereas recessive X-linked alleles (panel e) are more often exposed in males due to male hemizygosity. Y-linked genes (panel f) are only selected for male-specific effects.

Sex-biased or sex-limited inheritance, exhibited by the homogametic sex chromosomes, the heterogametic sex chromosomes and extra-nuclear genomes carried by mitochondria and chloroplasts, influences the effect of sex-specific selection. Contrasting the evolutionary signatures on sex chromosomes in particular with the autosomes is increasingly useful for uncovering the strength of sex-specific selection (Rice, 1984; Dean *et al*., 2012) (Fig.2).

Sexual conflict and the homogametic X and Z chromosomes are linked in several ways. First, the unique sex-specific selection pressures foster the nonrandom patterns of gene traffic to and from sex chromosomes for loci with sex-specific benefits (Zhang *et al*., 2010a,b). In addition to gene movement, sex-biased inheritance of the homogametic sex chromosomes suggests that they play a disproportionately large role in the evolution of sexual dimorphism via intralocus sexual conflict (Rice, 1984). The theoretical prediction that the X chromosome is both feminized and demasculinized, and the Z chromosome is masculinized and defeminized, is supported by numerous genomic analyses in animals (reviewed in Mank, 2009; Wright *et al.,* 2012), as well as plants (Spigler *et al*., 2011). Finally, sexual conflict may also foster the origin and/or turnover of sex chromosomes. In many clades, there is a high rate of sex chromosome turnover, which has been linked to sexual conflict (van Doorn & Kirkpatrick, 2007, 2010). The theoretical link has been supported by direct empirical evidence in cichlids, with the invasion of a novel sex determining locus (Roberts *et al*., 2009) as well as sticklebacks, with the origin of a neosex chromosome that contains loci for male courtship traits (Kitano *et al*., 2009; although see Natri *et al.,* 2013). This suggests a complex relationship between conflict and sex chromosomes, involving gene movement, the origin of new sex chromosomes, or gene expression changes on existing sex chromosomes.

Mating systems may also influence the degeneration rate of the heterogametic Y and W chromosomes. For sex chromosomes that evolve from existing autosomes, linkage between a sex determining locus and a nearby locus with sex-specific effects will result in selection to suppress recombination between the heterogametic and homogametic sex chromosome. As recombination suppression spreads across the heterogametic sex chromosomes, the Y and W chromosome–coding content degrades by neutral processes (Charlesworth, 2008). Additionally, the lowered effective population size of the heterogametic sex chromosome compared to the homogametic sex chromosome and the autosomes promotes degradation by background selection and hitchhiking. For relatively stable genes with low levels of gene translocation, the likelihood that a new sex determining gene will be located proximate to a sexually antagonistic locus may be roughly predicted by the proportion of loci in the genome that produce a sex-specific benefit. As mating system is one of the determinants controlling the proportion of the genome subject to sexually antagonistic selection, we might expect mating systems with high levels of conflict to result in faster spread of recombination suppression and therefore heterogametic sex chromosomes degeneration (Charlesworth & Mank, 2010).

Similar to the sex chromosomes, extra-nuclear genomes show sex-biased transmission patterns that influence the pattern of sex-specific selection. Mitochondria, although present and essential to both males and females, are only transmitted through the matriline and therefore, mitochondrial genomes are predicted to accumulate alleles beneficial to females but detrimental to males (Cosmides & Tooby, 1981; Frank & Hurst, 1996). A recent study supports this prediction, showing that the maternal transmission pattern results in a sieve that allows deleterious effects to persist if they are limited to males (Innocenti *et al*., 2011). This sex-specific sieve may act on other uniparentally inherited organelles, such as chloroplasts, thought the sieve effect likely varies greatly between dioecious and monecious species.

## Neutral sex-specific patterns

**Figure 3:**
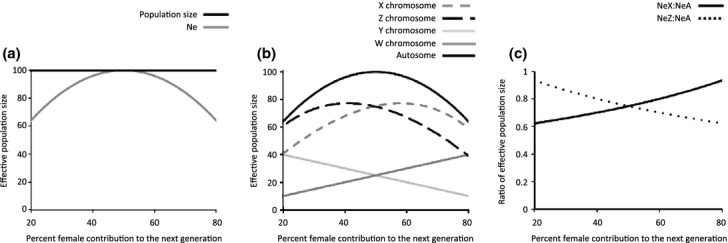
Mating systems and effective population size (N_E_). Increasing differences between male and female reproductive success reduces N_E_ (panel a), despite a constant overall population size. This difference between the sexes in reproductive success influences the N_E_ of different portions of the genome in different ways (panel b). For autosomal genes, the largest effective population size (N_EA_) is seen when the variance in male and female reproductive success is equal; however, N_EX_ and N_EW_ increase with a greater proportion of females than males contributing to the next generation. The opposite is seen for N_EZ_ and N_EY_, which are maximized when there are more males than females in the reproductive pool. This difference in the effect of mating system on the effective population size of different chromosomes makes contrasts between sex chromosomes and autosomes revealing (panel c).

Identifying signatures of genetic drift at the genetic level provides insight into the strength of neutral evolution. Mating systems define the variance in reproductive success between the sexes and thus the transmission of genetic material to subsequent generations, and the effect of mating system on the direction and rate of transmissions differs among regions of the genome. Although males and females share the autosomal portion of their genome equally, there is a pronounced asymmetry in the inheritance of the X chromosome (more often present in females), the Y chromosome (male-limited), the Z chromosome (more often present in males) and the W chromosome (female-limited). These inheritance patterns mean that different regions of the genome differ from each other in both their absolute effective populations size (N_E_), as well as their response in N_E_ to differences in male and female mating success (Fig.3). Drift for autosomal loci is minimized in monogamous species compared to other mating systems, and deviations from monogamy will lead to elevated rates of genetic drift and decrease the efficacy of selection across the genome as a whole (Hartl & Clark, 2006). However, the relationship between drift and selection plays out differently on the sex chromosomes. The effective population size of both the X and Z chromosomes (N_EX_ and N_EZ_) = ¾ that of the autosomes (N_EA_) in monogamous mating systems, creating a potential for increased genetic drift to act on homogametic sex chromosomes (Charlesworth *et al*., 1993; Vicoso & Charlesworth, 2009). Increased variance in male reproductive success associated with most forms of sexual selection increases N_EX_ and decreases N_EZ_ relative to N_EA_ (Fig.3b–c); therefore, sexual selection on males is predicted to increase rates of neutral evolution for Z chromosomes more than X chromosomes, termed Faster-Z and Faster-X evolution (Mank *et al*., 2010c). This is supported by some evidence from birds (Mank *et al*., 2007, 2010b), mammals (Lau *et al*., 2009) and *Drosophila* (Connallon, 2007; Singh *et al*., 2007; Baines *et al*., 2008); however, other data are discordant with the role of mating sytem and sex chromosome evolution (Haddrill *et al*., 2010).

These predictions for Faster-X and Faster-Z evolution are slightly altered under female promiscuity, primarily because the degree of variation in female reproductive success seen in polyandry is predicted to be less than the variation in male reproductive success seen in polygyny (Liker *et al*., 2001). Additionally, although female promiscuity can increase male-specific selection, it can also erode variance in male mating success (Collet *et al*., 2012).

The exact relationship between mating system and the strength of Faster-X or Faster-Z evolution is complicated by differences in the rate of recombination on the sex chromosomes, particularly the absence of recombination in *Drosophila* males, which raises the N_EX_ to near N_EA_, thus reducing the strength of drift (Connallon, 2007; Vicoso & Charlesworth, 2009). It is important to make clear that variance in male and female reproductive success affects all portions of the genome, however contrasting the rates of neutral evolution for different portions makes it possible to quantify the effect of mating system on genetic drift.

Mating system also affects the rates of evolution for the heterogametic W and Y chromosomes. N_EW_ and N_EY_ are both equal to 1/4 N_EA_ under monogamy, but sexual selection acting on males will decrease N_EY_ and increase N_EW_ compared to N_EA_. Although there are a host of factors affecting the evolution of heterogametic sex chromosomes, (reviewed in Charlesworth, 2008), the difference in N_EY_ and N_EW_ under sexual selection may accelerate the rate of degeneration of Y chromosomes compared to W chromosomes (Bachtrog *et al*., 2011). In addition to decay, changes in mating system may also result in accelerated rates of change and structural rearrangement in heterogametic sex chromosomes. Consistent with this, the Y chromosome is highly conserved between human, gorilla (Goto *et al*., 2009) and rhesus macaque (Hughes *et al*., 2012) but has shown rapid change in the intermediary chimpanzee lineage (Wilson & Makova, 2009; Hughes *et al*., 2010), and potential explanations for this rest on the promiscuous mating system observed in chimps. However, although the primate example fits the theoretical predictions, it is also anecdotal, and it is not yet possible to robustly test the relationship between mating system and Y chromosome degeneration. Additionally, the exact relationship between heterogametic sex chromosomes and mating system is complicated by several factors, including differential rates of gene acquisition (Koerich *et al*., 2008) and intrachromosomal recombination (Lange *et al*., 2009). Additionally, in some cases, recombination between the homogametic and heterogametic sex chromosome occurs in sex-reversed individuals (Stöck *et al*., 2011), which may act to prevent Y chromosome decay (Perrin, 2009).

## Conclusions and synthesis

Mating system can have profound effects on both neutral and adaptive genome evolution, and can also foster change in gene sequence, expression and post-translational modification for a large proportion of loci. At this point, the theoretical predictions linking mating system to genome evolution have been supported by many anecdotal species-specific studies. By utilizing recent advances in molecular genomics, many of these studies provide a long-term overview of the nature of sex-specific selection acting across thousands of genes over millions of years. However, this approach relies on a number of assumptions, namely that conflict is resolvable, which may not be the case for many loci. We also lack detailed information on the sex-specific fitness effects of certain genomic mechanisms thought to be shaped by sex-specific selection. Phenotypic studies can provide much needed information on sex-specific fitness optima and the consequences of deviating from these optima. We therefore emphasize the need for a combined approach, incorporating phenotypic studies with genomic data in order to examine the nature of sexual conflict currently acting on the genome and the fitness consequences of the genomic mechanisms thought to resolve sexual conflict.

Furthermore, recent developments in next generation sequencing methods now facilitate studies utilizing the variation in mating system across a wide range of species. Currently, we lack a holistic, robust and quantitative understanding of the relationship between mating system and genome evolution and a cohesive picture of how the myriad of sex-specific forces integrate with one another. This robust, quantitative and cohesive understanding requires comprehensive studies in clades with a range of mating systems. Although model organisms can be useful, the most relevant behavioural ecologies for this type of analysis are often associated with nonmodel organisms. A comparative approach across a range of mating systems is much needed to shed further light on the nature of sex-specific selection acting on the genome.

## Abbreviations

Fast -X Effect (Fast-Z Effect): Higher ratio of nonsynonymous to synonymous substitutions for X- and Z-linked genes compared to autosomal genes, due to a combination of hemizygous exposure of mutations in the heterogametic sex and lowered effective population size.
Hemizygosity: Having unpaired chromosomes in a diploid cell. The heterogametic sex (males in XY systems and females in ZW species) has only one copy of each sex chromosome and is therefore hemizygous for the sex chromosomes.
Heterogametic sex chromosomes: The sex chromosomes that are sex-limited in the heterogametic sex, namely the Y in male heterogamety and the W in female heterogamety.
Homogametic sex chromosomes: The X chromosome in male heterogamety and the Z in female heterogamety.
Interlocus sexual conflict: Type of antagonism resulting when sexual conflict plays out over two or more loci. This often results in an unresolvable arms race between females and males.
Intralocus sexual conflict: Type of antagonism resulting when male and female optima differ for a single locus. Intralocus sexual conflict can be resolved via a variety of genomic mechanisms.
Monogamy: Mating system where males and females pair and are sexually exclusive.
Polyandry: Mating system where females have more than one mate at any given time, resulting in greater variance in female compared to male reproductive success.
Polygamy: General term describing a deviation from monogamy, encompassing both polyandrous and polygynous mating systems.
Polygyny: Mating system where males have more than one mate at a time, resulting in greater variance in male compared to female reproductive success.
Polyspermy: A condition resulting when more than one sperm cell fertilizes an ovum, often resulting in inviability.
